# Physico-Chemical and Biological Evaluation of PLCL/SF Nanofibers Loaded with Oregano Essential Oil

**DOI:** 10.3390/pharmaceutics11080386

**Published:** 2019-08-02

**Authors:** Atta ur Rehman Khan, Muhammad Nadeem, M. Aqeel Bhutto, Fan Yu, Xianrui Xie, Hany El-Hamshary, Ayman El-Faham, Usama A. Ibrahim, Xiumei Mo

**Affiliations:** 1 College of Chemistry, Chemical Engineering & Biotechnology, Donghua University, Shanghai 201620, China; 2 Institute of Biotechnology and Genetic Engineering, University of Sindh, Jamshoro 76080, Pakistan; 3 Chemistry Department, College of Science, King Saud University, P.O. Box 2455, Riyadh 11451, Saudi Arabia

**Keywords:** oregano essential oil, electrospun nanofibers PLCL, SF, drug release, mechanical properties, antioxidant activity, anti-tumor activity

## Abstract

Essential oils are complex volatile compounds, extracted from specific plant species, with promising therapeutic potentials. However, their volatile nature presents a major hindrance in using them as therapeutic agents. In the current study, we successfully encapsulated oregano essential oil (OEO) into Poly (l-lactic acid-*co*-e-caprolactone) /Silk Fibroin (PLCL/SF) polymers through electrospinning. The nanofibrous membrane (NF) was fabricated and characterized for various physico-chemical and biological attributions. Homogenous and bead free morphology was confirmed by scanning electron microscopy (SEM). Attenuated total reflection Fourier transform infrared spectroscopy (ATR-FTIR) confirmed the successful loading of OEO and its physical interaction with the blend of PLCL/SF. Moreover, thermogravimetric analysis (TGA) also confirmed the successful loading and thermostability of the OEO. Although a significant change was noted in tensile strength due to the loading of OEO, the mechanical behaviour still falls into the acceptable ranges required for skin tissue engineering. Similarly, fabricated material was evaluated for its biological significance. Liquid chromatography-mass spectrometry (LC-MS) was employed to determine the release behaviour of OEO from electrospun membranes. LC-MS data, noted for 48 h, confirmed the biphasic release of OEO. Furthermore, NF membranes have shown strong antioxidant and anti-tumor activities. This material is promising and can be implanted to avoid the recurrence of the tumor after its surgical removal.

## 1. Introduction

Essential oils (EO) are a mixture of volatile compounds produced by aromatic plants [1,2]. There are various methods to extract these compounds, including steam distillation as the major one [3]. A great variety of plants have been explored for their EO content. Within the family of these aromatic plants, *Oreganum vulgare* L. is known for its essential oil, termed as oregano essential oil (OEO), a famous compound with established antioxidative and antimicrobial activity [4,5]. These activities can mainly be attributed to two phenols; carvacrol and thymol (major components of oregano essential oil constituting more than 80% of total) and the monoterpene hydrocarbons *p*-cymene and γ-terpinene, although only present in traces [6]. The detail Gas chromatography-Mass spectrometry (GC/MS) report of OEO, as used in the experiment, can be accessed on the manufacturer website [7]. Carvacrol, a major component, has been extensively analyzed for its bioactivities including anti-oxidant, antimicrobial, and anti-cancer. This compound was found potentially useful in tackling free radical-mediated injuries and saves DNA damage due to its ability to increase the level of anti-oxidant along with anti-lipid peroxidative activity [8,9]. These properties can be associated with a beneficial impact during various pathological conditions, including cancer.

However, the instability of this oil, like other essential oils, has remained a major hindrance to its use as a therapeutic agent. Fortunately, electrospinning is an emerging technology which provides an option to preserve the bioactivity of such valuable compounds by converting them into a fibrous scaffold [10]. These resultant fibrous membranes not only provide encapsulation to these bioactive compounds but also maintain and enhance their therapeutic potential by providing a large surface area to volume ratio. For electrospinning, the major task is to identify and use a suitable polymer system which can stabilize the compound as well as exhibit the necessary physico-chemical behaviour required to accomplished desired tasks.

Silk fibroin (SF) is a natural protein that is extensively used in tissue engineering due to its good biocompatibility, oxygen and water vapor permeability, biodegradability, and lower inflammatory response than collagen. It’s easy availability and cost-effectiveness are other added advantages [11,12]. However, the weak mechanical property of regenerated SF is a major issue for which it needs to be blended with some other polymer that possesses good mechanical strength. Depending upon the applications, various synthetic polymers can be blended with SF to compensate for its weak mechanical property. Poly (l-lactic acid-*co*-e-caprolactone) (P(LLA-CL)) is a copolymer of poly (L-lactic acid) (PLLA) and polycaprolactone (PCL) possessing good mechanical strength. Moreover, its biocompatibility and biodegradability are also features that make this polymer a suitable candidate for tissue engineering [13]. The mixture of these two polymers can result in a fibrous structure possessing good mechanical, physical, and chemical properties [14].

Few studies have been carried out regarding exploitation of the bioactivity of essential oils (EOs) using the electrospinning technique to gain fibrous scaffolds required for various applications. A literature review reveals that some EOs have been successfully electrospun by using polymers as a carrier and encapsulating agent. Until now, the EOs which have been electrospun include Candeia [15], cinnamon [16,17], lemongrass and peppermint [16], tea tree [18], thymol [19], lavender [20], orange essential oil [21], Ginger [22], Eugenol [23] and Chamomile [24]. Although many studies have explored the therapeutic potential of OEO, no one has reported its applications in nanofibrous mats. In a study, Hossseini et al. attempted to stabilize OEO through encapsulation in chitosan nanoparticles [25]. Other studies were also conducted to observe its antimicrobial potential [26,27,28], anti-inflammatory, [29] anticancer, [30,31,32,33], and antioxidant activities [4,34]. However, the electrospinning of OEO, along with some polymer system, to encapsulate it into nanofibers cannot yet be achieved. These scaffolds can be used for many biomedical applications including anti-tumor, anti-oxidant, antibacterial, anti-inflammatory, and wound healing activities.

The present research focused on the fabrication and characterization of nanofibers loaded with OEO. PLCL and SF were chosen as a polymer system for the encapsulation of OEO. In this study, we have electrospun OEO along with the blend of PLCL/SF to achieve a nanofiber structure. These fibers were subjected to physico-chemical and biological characterizations. The morphology of the fibers was determined by SEM. FTIR was employed to assess the interaction of OEO with polymers. The fabricated material was also analyzed for thermal properties through TGA. Tensile strength, Young’s Modulus, and elongation at break were calculated to assess the mechanical changes due to loading OEO. Drug release was studied through LC-MS. The fabricated material was also assessed for its therapeutic potential. A DPPH assay was used to measure the anti-oxidant activity of the material. Furthermore, the anti-proliferative activity of the fabricated material was assessed against the 4T1 cell line through in-vitro cell culture.

## 2. Material and Method

### 2.1. Material

The co-polymer of poly (l-lactide-*co*-caprolactone) (PLCL) (50:50) was purchased from Jinan Daigang Bioengineering Co. Ltd. (Jinan, China). Cocoons of *Bombyx mori* silkworm were kindly supplied by Jiaxing Silk Co. Ltd. (Jiaxing, China) and 1,1,1,3,3,3,-hexafluoro-2-isopropanol (HFIP) was purchased from Daikin Industries Ltd. (Osaka, Japan). Oregano essential oil (OEO) (100% pure), extracted from *Origanum vulgare,* through steam distillation, was purchased from Edens Garden, 1322 Calle Avanzado, San Clemente, CA 92673, USA. L-Ascorbic Acid and 2,2-diphenyl-1-picryl-hydrazyl-hydrate (DPPH) was obtained from Sinopharm Chemical Reagent Co., Ltd. (Shanghai, China) 4T1 cell line was obtained from the Typical Culture Collection Committee Cell Bank, Chinese Academy of Science. Cell counting Kit-8 (CCK-8) 2-(2-methoxy-4-nitrophenyl)-3-(4-Nitphenyl)-5-(2,4-disulfophenyl)-2*H*-tetrazole monosodium salt) and phosphate-buffered saline (PBS) (pH 7.2–7.4) were purchased from Beijing Solarbio Science & Technology Co., Ltd. (Beijing, China) Cell culture media including RPMI (1640), Fetal Bovine Serum (FBS), and related media reagents were obtained from Gibco Life Technologies, Co. Waltham, MA, USA.

### 2.2. Preparation of Regenerated Silk Fibroin (SF)

The regenerated silk fibroin (SF) was prepared by following the earlier published procedure [35]. Briefly, raw silk was degummed three times with 0.5% (*w/w*) Na_2_CO_3_ solution at 100 °C for 30 min and washed with distilled water. The degummed silk was dissolved in CaCl_2_/H_2_O/EtOH solution (molar ratio 1:8:2) at 70 °C for 1 h. The solution was dialyzed using cellulose tube (250-7u; Sigma, Saint Louis, MO, USA) in distilled water for three days at room temperature. Distilled water was replaced with fresh water every after 6 h. Finally, the SF solution was filtered and freeze-dried to obtain the regenerated SF.

### 2.3. Nanofiber Fabrication

#### 2.3.1. Electrospinning

The electrospinning solution was prepared by dissolving the PLCL and SF with the weight ratio of 70:30 (PLCL/SF) in 1,1,1,3,3,3-Hexafluoro-2-isopropanol (HFIP) to obtain 10% (*w/v*) final polymer concentration. Moreover, three other solutions were made to incorporate various concentration of OEO; 2.5%, 5%, and 7.5% (*v/v*) into PLCL/SF solutions. All prepared solutions were kept on a stirrer overnight. The 10 mL of each solution was pumped through a 21 gauge needle with a flow rate of 1 mL/h whereas the distance between the metal plate collector and syringe was kept in between 12–14 cm. A high voltage of 12–15 kV was supplied by a high voltage power supply (BGG6-358, BMEI Co Ltd., Beijing, China). Humidity and temperature were also kept constant at 50% and 25 °C, respectively, during the spinning of various established groups of solutions.

#### 2.3.2. Crosslinking

Each membrane, just after electrospinning, was placed in a sealed desiccator containing glutaraldehyde vapours (evaporating from 10 mL of 25% glutaraldehyde aqueous solution) at room temperature for 3 h. After treating with glutaraldehyde vapours, the membranes were shifted to a vacuum oven and dried for three days prior to characterization.

### 2.4. Physico-Chemical Characterization

#### 2.4.1. Morphology of Electrospun Nanofibers

The structural morphology of the prepared nanofibers was observed by Scanning Electron Microscope (SEM, TM-100; Hitachi, Tokyo, Japan). Dry samples were sputter-coated with gold for 45 s before loading into SEM. The observation was carried out under the accelerating voltage of 10 kV. Finally, according to the SEM image, 100 nanofibers were randomly selected, measured, and contrasted using Image J software (National Institute of Health, Bethesda, MD, USA) to calculate the average diameter distribution and fiber diameter.

#### 2.4.2. Pore Size and Porosity

The pore size of the electrospun nanofiber membranes was measured using Image J software based on the SEM images. All NF membranes, tested for porosity and pore size, were obtained after 1 h continuous spinning with the flow rate of 1 mL/h. One hundred pore diameters on different parts of the SEM images were selected randomly to measure the pore size distribution. The porosity of the prepared samples was determined by the reported method [36]. Briefly, the nanofibrous membranes were cut into a rectangular shape and the volume was measured. These membranes were immersed in absolute ethanol until saturation. The nanofibrous membranes were weighed before and after the immersion in alcohol. The porosity was calculated using the formula; P = (W_2_ − W_1_)/(ρV_1_). Where W_1_ and W_2_ indicate the weight of the electrospun membrane before and after immersion respectively, V_1_ is the volume before immersing and ρ is a constant of the density of alcohol. All samples were used in triplicate in this measurement.

#### 2.4.3. Attenuated Total Reflection Fourier Transform Infrared Spectroscopy (ATR-FTIR)

ATR-FTIR spectra of pure OEO, SF, PLCL, and electrospun NF membranes containing 0%, 2.5%, 5%, and 7.5% OEO were obtained in absorption mode at 2 cm^−1^ intervals in the range of 500–4000 cm^−1^ using an ATR-FTIR spectrophotometer (Thermo Nicolet Avatar 380, Thermo Scientific, White Bear Photonics, White Bear Lake, Minnesota, USA).

#### 2.4.4. Mechanical Properties

The mechanical properties of the nanofibers were characterized by a Universal Materials Testing Machine (H5K-S, Hounsfield, UK) with the ambient temperature of 20 °C and humidity of 65%. All specimens (50 mm × 10 mm, n = 5) were tested with a cross-head speed of 10 mm min^−1^ until breakage. The samples for each group were tested both in a dry and wet state. The specimen thickness was measured using a digital gauge meter, having a precision of 1μm. For the wet state, the samples were immersed in the Phosphate-buffered saline (PBS) overnight. The tensile strength, elongation at break and Young’s modulus were calculated.

#### 2.4.5. Thermogravimetric Analysis (TGA)

The thermodynamic properties of the electrospun membranes of pure SF and PLCL were determined by thermogravimetry (TG) using TG 209 F1 Libra Thermogravimetric Analyser, Netzsch, Germany. Weight of the samples was measured into an Al_2_O_3_ (aluminium oxide) crucible and the profiles were recorded from room temperature to 900 °C under a nitrogen atmosphere at a heating rate of 10 °C/min.

#### 2.4.6. Invitro Drug Release Study

The release profile of OEO loaded PLCL/SF NF membranes was investigated through liquid chromatography-mass spectrometry (LC-MS). The release medium, used in a previous experiment, consisted of 60% PBS and 40% ethanol [25]. All drug-loaded samples (50 mg) were incubated at 37 °C in a 4 mL vial containing the above-mentioned release medium under stirring conditions. Aliquots of samples (1 mL) were taken from the release medium after specific time intervals and the same volume was replaced with fresh media to maintain the sink condition. The amount of OEO released at various times, up to 48 h, was determined using an LC-MS technique. The calibration curve of OEO was established, and the cumulative percentage of OEO release was calculated and plotted versus time according to the following equation.

(1) $$\mathrm{Cumulative}\;\mathrm{Drug}\;\mathrm{Release}\;\left(\%\right)=\frac{\mathrm{Drug}\;\mathrm{release}\;\mathrm{at}\;\mathrm{each}\;\mathrm{time}\;\mathrm{interval}}{\mathrm{Total}\;\mathrm{drug}\;\mathrm{Loaded}}\times \;100$$

#### 2.4.7. Liquid Chromatography-Mass Spectrometry (LC-MS)

##### Instrumentation

All medium samples were analyzed on LC-MS-2020 (Shimadzu, Kyoto, Japan) equipped with a UV and MS detector range from 200 nm to 600 nm and two pumps used for the elution of the mobile phase solution. LC solution software was used for data acquisition. The separation was performed on the C18 column (100 × 2.1 mm id × 5 μm particle diameter) with security guard C18 4 mm × 3 mm id (Phenomenex, Torrance, CA, USA). Calibrated automatic pipettes and analytical balance were used for the volume and weight measurement.

##### Chromatographic Conditions

All the mobile phase solvents were processed in an ultrasonic bath for 25 min for sonication before analysis. The analysis was performed on the C18 column I.D 2.1 × 100 mm with mobile phase acetonitrile 50% (A) and Water 50% (B). The injection volume of the sample was 10 μL, and the flow rate was kept at 0.3 mL/min. The column temperature was kept at 35 °C, and a UV wavelength of 275 nm was used for the detection of OEO contents in the media. The isocratic method was used for the elution of the sample.

##### Preparation of Standard and Stock Solution

The standard solution of pure OEO 1000 mg/L was prepared by the addition of 100 mg of pure OEO in 100 mL flask. Afterward, a solution with 70% methanol and 30% ultrapure water, obtained from Milli-Q, was added to achieve the final volume up to 100 mL and stored in a refrigerator at 4 °C. Working standard solution of 4, 8, 16, 32 and 64 mg/L for calibration curve was freshly prepared before starting the analysis from the stock solution. The correlation efficient r^2^ of the calibration curve was 0.9986 throughout the experiment.

### 2.5. Biological Evaluation

#### 2.5.1. Antioxidant Activity

To determine antioxidant activity, a 2,2-diphenyl-1-picryl-hydrazyl-hydrate (DPPH) assay was employed according to the prescribed method [37]. First, 0.3 mM of DPPH radical solution was prepared, and samples including pure OEO, ascorbic acid and NF membranes (10 mg weight each) were mixed and put in the dark at room temperature. Both concentration and time-dependent antioxidant activity were determined. Pure ascorbic acid (AA) was taken as a standard antioxidant agent. Nine different groups were selected for the determination of antioxidant activity. These groups include: pure OEO (1,2 mg), pure ascorbic acid (1,2 mg), water, PLCL/SF, PLCL/SF 2.5% OEO, PLCL/SF 5% OEO and PLCL/SF 7.5% OEO.

#### 2.5.2. Anti-Tumor Activity

The anti-tumor activity of NF membranes loaded with different formulations of OEO was tested against mammary carcinoma (mouse) 4T1 cell line. PLCL/SF was taken as a negative control. Total cell potential (TCP) was also tested without using the NF membrane. The 4T1 cell line was cultured in the RPMI media supplemented with 10% FBS and 1% penicillin-streptomycin formulation at 37 °C with 5% CO_2_ in a humidified atmosphere. The medium was changed after 48 h. When cells reached over 80% confluency, the adhered cells were detached with the help of 0.25% trypsin-EDTA and counted by using an automatic cell counter. Prior to this, 14 mm of NF membrane were cut and sterilized under UV light overnight. Before seeding cells, NF membranes were washed with phosphate buffer saline (PBS). Subsequently, 24 well culture plates were taken, and NF membranes were fixed with stainless steel rings. About 10,000 cells suspended in 500 µL culture media per well were seeded and three samples were tested for each group. Cell viability was measured with the help of cell counting kit-8 (CCK-8) assay. Cells were incubated on material for 24, 48, and 72 h. The CCK-8 solution was made in pure RPMI (without FBS) with 1:9. About 300 µL CCK-8 solution was put in each well and samples were placed in an incubator for 2 h. After that, absorbance was noted at 450 nm/630 nm using a microplate reader. The presence of viable cells was also confirmed by using a Calcein assay. Media was removed, and cells were washed with PBS. Then, 400 µL of Calcein solution (Calcein + PBS) was added and incubated at 37 °C for 30 min. Absorbance was noted at 485 nm/535 nm using a microplate reader.

##### Statistical Analysis

All the quantitative data were expressed as a mean ± standard deviation (SD). The statistical analysis was carried out using one-way ANOVA and a value of *p* < 0.05 was considered statistically significant.

## 3. Result and Discussion

### 3.1. Morphology of the Electrospun Nanofibers

Electrospinning is a facile technique to fabricate nanofibrous structures which mimic those of native extracellular matrix (ECM) with a high porosity and surface area. The NF are extensively used in tissue engineering applications [38]. Various electrospinning parameters, including solution concentration, surface tension, viscosity, humidity, temperature, electrical conductivity, flow rate, the distance between collector and syringe, etc. affect the fiber morphology and diameter [39,40]. Additionally, the spinnability of the electrospinning solutions and the nanofiber morphologies are highly influenced by the solvent used to make the spinning solution [41,42]. In the present study, HFIP was used as a solvent to dissolve both polymers (SF and PLCL) and the drug (OEO).

**Figure 1:**
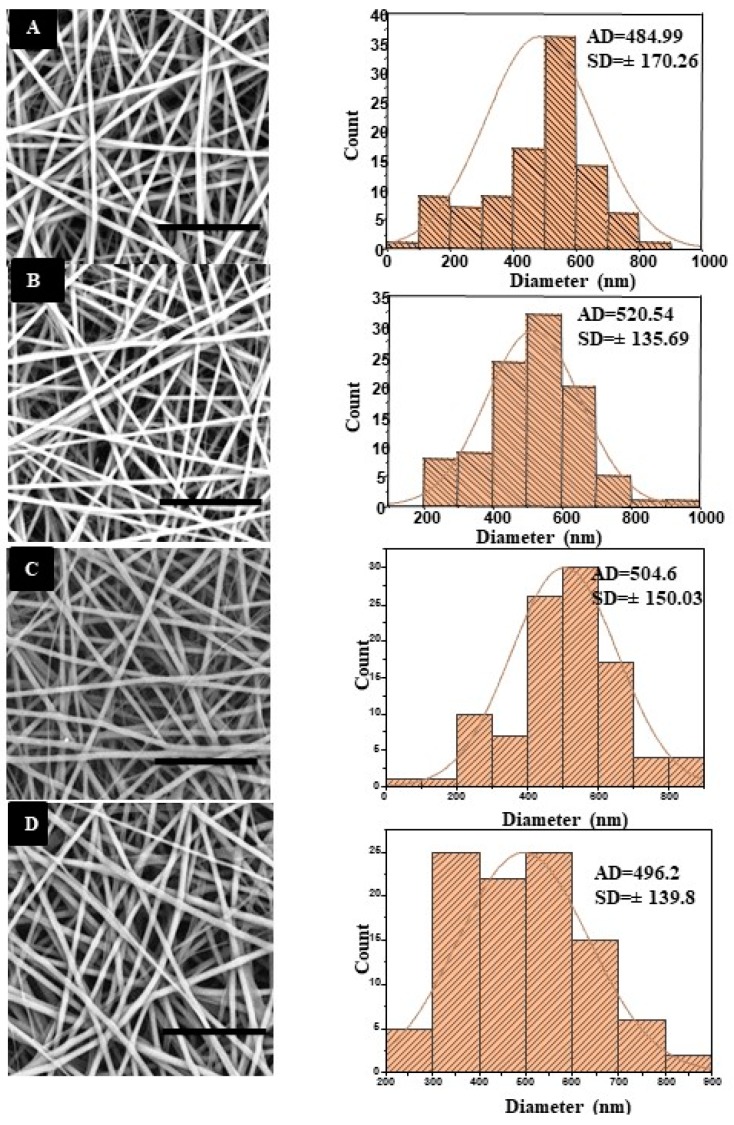
**Scanning Electron Micrograph** (SEM) (Right) and histogram (Left), (scale bar = 20 µm), (**A**) Poly (l-lactic acid-*co*-e-caprolactone)/Silk Fibroin (PLCL/SF), (**B**) PLCL/SF/2.5% Oregano Essential Oil (OEO), (**C**) PLCL/SF/5% OEO, (**D**) PLCL/SF/7.5% OEO.

The morphology and diameter distribution of the electrospun nanofibers are shown in Figure 1. SEM images showed that the average diameter of PLCL/SF nanofiber was 484.99 ± 170.26 nm (Figure 1A) and PLCL/SF/2.5% OEO was 520.54 ± 135.69 nm (Figure 1B). However, diameters of the PLCL/SF/5% OEO and PLCL/SF/7.5% OEO were noted as 504.6 ± 150.03 nm (Figure 1C) and 496.2 ± 139.8 nm (Figure 1D), respectively. From the SEM images, it can be deduced that smooth and bead free fibers were obtained and there was no significant effect of the addition of OEO on the morphology of the NF. No phase separation of oil was seen which indicates that OEO has a stable interaction with PLCL/SF polymers. It also indicates that OEO was completely dissolved in the polymer solution, which justifies the blend electrospinning carried out in this experiment. Statistical analysis (one-way ANOVA) of the data showed that the difference among fiber diameters of various groups remained insignificant (*p* < 0.05).

### 3.2. Pore Size and Porosity

Mean pore diameter and porosity of the fabricated material are shown in Table 1. Although the mean pore diameter is different in slight proportion however, this difference is statistically insignificant (*p* < 0.05). However, a small difference in average pore diameter can be related to an average increase in fiber diameter which increased with increasing drug contents.

Fiber diameter plays a key role in controlling the pore diameter of the NF [43]. The porosity data revealed that all NF membranes possessed porosity range between 50–70%. The results, as shown in Table 1, indicate an increase in porosity with increasing drug concentration and this can be attributed to the drug present on the surface, which swiftly dissolves into the medium resulting in higher porosity. Statistically (*p* < 0.05), the average porosity of PLCL/SF/2.5% remained insignificant compared to PLCL/SF, whereas PLCL/SF/5% and PLCL/SF/7.5% remained significantly different from the control because of the higher drug contents attached on the surface. This high porosity is a desirable feature which helps in transferring nutrients and oxygen to the inner surface. Moreover, a high porosity also involves absorbing wound exudates from the wound surface, which reduces the chances of infection [36,44].

### 3.3. Attenuated Total Reflection Fourier Transform Infrared Spectroscopy (ATR-FTIR)

**Figure 2:**
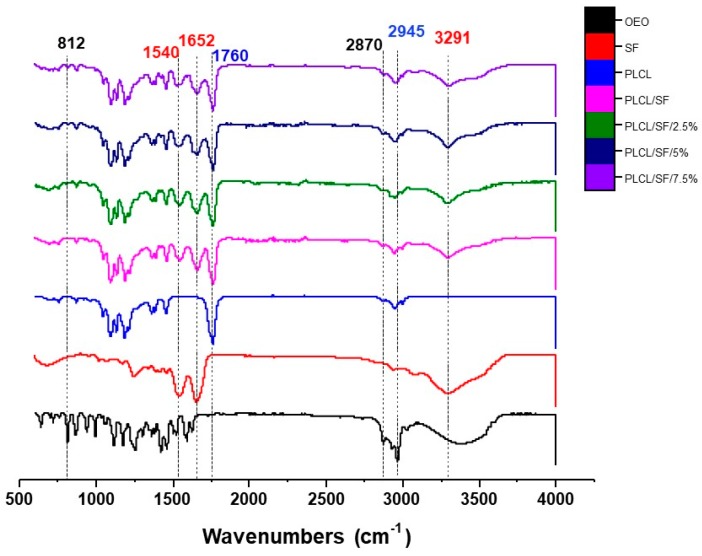
Attenuated total reflection Fourier transform infrared spectroscopy (ATR-FTIR) spectra of pure OEO, SF, PLCL and the electrospun Nano Fibrous membranes with 0%, 2.5%, 5%, and 7.5%) OEO.

ATR-FTIR spectra of the various samples of the NF membrane indicated with different colours is shown in Figure 2. OEO showed two distinct peaks by which the oil contents can be traced in the electrospun membrane. One peak belongs to the functional group region and appeared around 2870 cm^−1^. This peak corresponds to the HO-C stretch in the carboxyl group. The other characteristic peak appeared in the fingerprint area around 812 cm^−1^, confirming the presence of OEO. This peak is associated with ring vibration in carvacrol (A major component of OEO) [45]. In FTIR, these bands are intense and can be attributed to an out of plane C-H wagging vibration, a significant signal used to distinguish different types of aromatic ring substitutions [46].

The characteristic peaks for the SF appeared around 1652 cm^−1^ and 1540 cm^−1^. Protein material shows characteristic vibration bands at 1630~1650 cm^−1^ for amide I (C=O stretching), at 1520~1540 cm^−1^ for amide II (C=O stretching). For SF these peaks appeared around 1652 cm^−1^ and 1540 cm^−1^ corresponding random coil for amide I amide II respectively [47]. Another intense band appeared around 3291 cm^−1^, corresponding to N–H and O–H stretching and confirming the presence of SF. For PLCL, the peak appeared around 1760 cm^−1^ which is specific to C–O–C stretching vibration [13]. This is the characteristic peak in the samples containing either pure PLCL or PLCL combined with SF and drug. Another distinctive peak related to PLCL appeared around 2945 cm^−1^ which is due to the stretching vibration of –CH_2_ and the vibration of the –C=O bond [48]. No chemical interaction was seen among combining substances because no new peak appeared or significantly shifted. The nature of the interaction was physical among these three substances.

### 3.4. Mechanical Properties

The NF membrane designed for biomedical applications, especially wound healing, should provide tensile strength as well as flexibility in both dry and wet states for better clinical operation and handling. All the NF membranes (both dry and wet state) were in an acceptable range of tensile strength and elongation at break required for skin tissue engineering. According to the literature human skin varies in the range of 1–32 MPa for tensile strength and 17–207% for elongation at break. However, certain factors like age, skin colour, and genetic heritage can lead to the heterogenicity in human skin [49].

**Figure 3:**
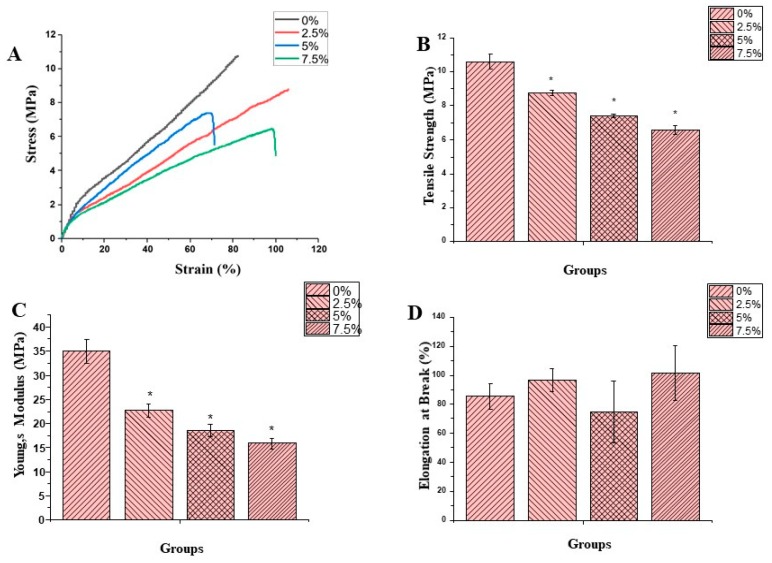

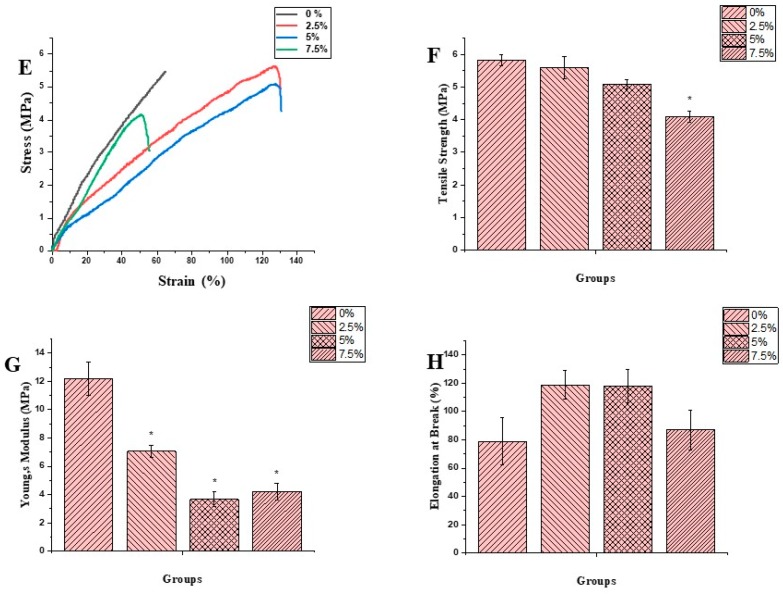
Stress-Strain curve, Tensile Strength, Young’s Modulus and Elongation at break for Dry Figure 3 (**A**–**D**) and Wet Figure 3 (**E**–**H**) respectively. (* = significance difference in comparison with control).

Figure 3A–D shows the mechanical properties of the fiber membrane in dry form. According to the results shown in Figure 3B, tensile strength of NF membranes with 0%, 2.5%, 5% and 7.5% OEO contents remained 10.61 ± 0.42, 8.76 ± 0.16, 7.40 ± 0.10 and 6.56 ± 0.28 respectively. All the OEO containing NF membranes exhibited tensile strength significantly different (*p* < 0.05) form control and decreased with increasing oil contents. The tensile strength of the PLCL/SF agrees with the previous work [14]. The decrease in tensile strength can be attributed to the oil contents attached to the surface resulting in a decrease in fiber strength. Moreover, porosity also increased with increasing oil contents which resulted in a relatively low tensile strength. This is probably the first study in which the incorporation of oil into the fiber membrane is studied in a mechanical point of view. However, all membranes fall in an acceptable range of mechanical strength required for skin tissue application. Figure 3C shows the Young’s modulus of the various membranes. The results indicated that values for the Young’s Modulus remained 34.94 ± 2.47, 22.71 ± 1.34, 18.53 ± 1.16, and 15.82 ± 1.02 for 0%, 2.5%, 5%, and 7.5%, respectively. Statistical analysis (*p* < 0.05) showed that all the oil containing fiber membranes are significantly different from the control. This decrease in Young’s Modulus can be linked to the decrease in tensile strength and an increase in the elasticity of the membrane due to the incorporation of oil into the NF membrane. The elongation at break of the dry samples are shown in Figure 3D. According to the data, the values for elongation at break remained 85.4 ± 8.7, 96.5 ± 8.1, 74.8 ± 21.5, and 101.3 ± 19.1 for 0%, 2.5%, 5%, and 7.5%, respectively. Although we can see some variation among various groups however, these variations are statistically insignificant. In general, OEO contents produced elasticity in the NF membranes.

Figure 3E–H shows the typical stress-strain curve, tensile strength, Young’s modulus and elongation at break of the membranes in the wet state. The mechanical strength of the NF membrane was tested by immersion in PBS overnight, considering the wound environment. The result indicated the considerable mechanical strength even after immersing in PBS. According to the results, tensile strength for the membranes remained 5.82 ± 0.17, 5.58 ± 0.33, 5.08 ± 0.13, and 4.07 ± 0.16 for 0%, 2.5%, 5%, and 7.5%, respectively. Whereas the values for the Young’s Modulus remained 12.19 ± 1.18, 7.05 ± 0.40, 3.65 ± 0.52, and 4.20 ± 0.57 for 0%, 2.5%, 5%, and 7.5%, respectively. The elongation at break also remained under an acceptable range. However, in comparison to the dry state, this strength is significantly low. Similarly, the Young’s Modulus also remained proportionally lower compared to the dry state.

### 3.5. Thermogravimetric Analysis (TGA)

**Figure 4:**
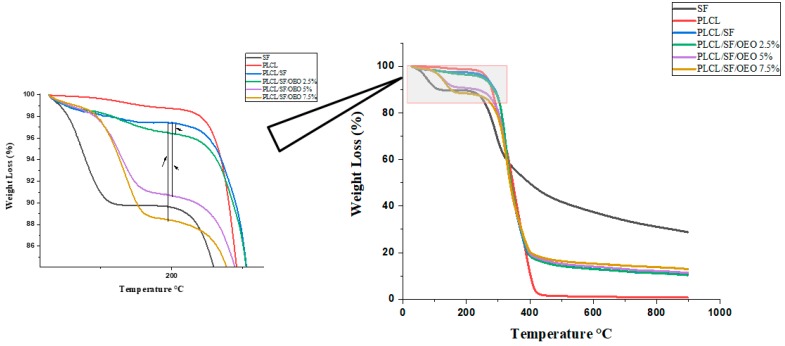
Thermogravimetric Analysis (TGA) graph of pure SF, PLCL and electrospun NF membranes with OEO (0%, 2.5%, 5% and 7.5%).

TGA is a useful technique to study the weight change of a sample as a function of temperature and to assess the thermal stability of the sample. Figure 4 displays the mass loss (%) as a function of temperature. TGA was carried out for six different samples. These include pure SF and PLCL, electrospun membranes of PLCL/SF, PLCL/SF/2.5% OEO, PLCL/SF/5% OEO, and PLCL/SF/7.5% OEO. The detail mass loss in relation to the temperature is shown in Table 2. According to a previous study, OEO showed one step of mass loss starting at 171 °C (peak at 195 °C) [25]. The thermal degradation of pure silk showed two-phase transitions of mass loss. One mass loss started around 50 °C and a steep loss continued up to 120 °C. An approximately 8.3% mass loss was noted during this range. This mass loss can be attributed to the removal of structural water during the initial heating. Afterward, it showed stability up to 244 °C and only about 2.6% mass loss was noted. The second stage of mass loss started from 244 and continued up to 600 °C. During this temperature range, about 55% of the mass loss was evident in which major bulk can be seen up to 350 °C. These transition phases generally agree with the previously available data [50]. On the other hand, PLCL showed only one phase of weight loss due to lack of bound water with the structure. The degradation phase started from 245 °C and continued up to 436 °C. In the previous study [50], the degradation temperature of PLCL remained 298 °C. This difference can be attributed to the structural variation of PLCL because it is a copolymer of PLA and PCL with different mass ratios. In our study, we used a 50:50 mass ratio. Resultantly, the structure was more elastic. The four groups shown in Figure 4 are electrospun membranes with various mass ratios of OEO and one without OEO. By comparing the TGA of membranes (with OEO) to the membrane (without OEO), we can analyse the impact of OEO on the thermal properties of the membrane. The data obtained was divided into four distinct phases of mass losses and compared. These phases were 100–250 °C, 250–400 °C, 400–452 °C, 452–600 °C. The temperature ranges were kept constant for comparative purpose. In the temperature range of 100–250 °C, the total mass loss of PLCL/SF remained 1.24%. This shows that SF got thermal stability by blending with PLCL. The temperature range of 250–400 °C can be attributed to a major mass loss of both PLCL and SF. The membrane with 2.5% OEO showed a loss of 3.5% within the temperature range of 100–250 °C. The additional mass loss of about 2.5% can be attributed to the loss of OEO from the membrane. This not only confirms the successful loading of OEO during electrospinning, but also confirms that OEO got more thermal stability in a polymer system than pure. In the same range of temperature, the mass loss for 5% and 7.5% OEO loaded NF remained 8.5 and 10.7% confirming the higher mass loss of OEO. The remaining data reveal that OEO did not affect the overall thermal properties of the PLCL/SF membrane.

### 3.6. Invitro Drug Release Study

**Figure 5:**
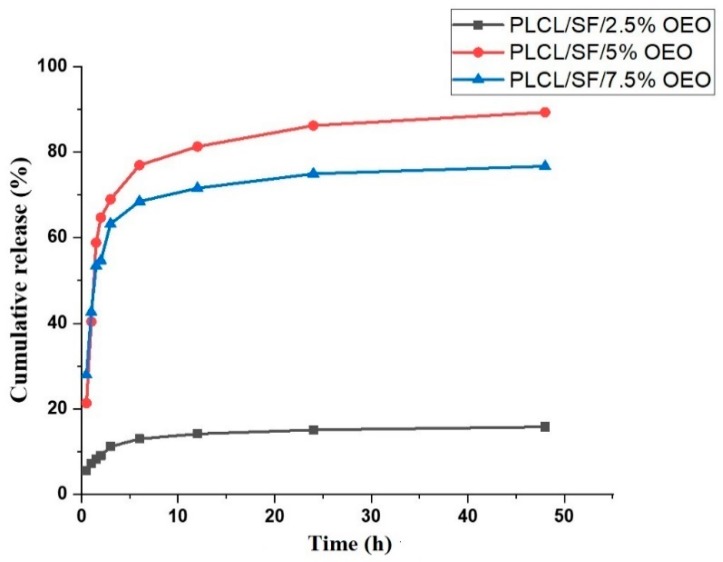
Cumulative release (%) of OEO from PLCL/SF/2.5% OEO, PLCL/SF/5% OEO and PLCL/SF/7.5% OEO.

Figure 5 shows the cumulative release behaviour of NF membranes loaded with various OEO concentrations. Data reveals that only 5.6% of the total drug was released in the first 30 min in case of NF membrane loaded with 2.5% OEO concentration. This indicates the low amount of OEO attached with the surface and complete encapsulation of OEO into the fiber matrix. Later, a steady release was observed. This steady release continued for 6 h followed by sustained drug release.

NF membrane containing 5% OEO showed a biphasic release behaviour; a burst release in the first three h in which a cumulative release of about 77% was observed followed by steady release up to 48 h. After 48 h, the total drug released estimated up to 89%. The NF membrane with 7.5% OEO showed the same biphasic release behaviour. An initial burst release was observed within the first three h in which total 68% drug was released. Later, a steady release was observed which lasted up to 48 h. This biphasic release of oil is largely in agreement with the release behaviour of OEO studied in a reported work [25]. It is reported that the mechanism of the drug release behaviour can be diffusion, polymer erosion, disintegration, and desorption, or a combination of any of these [51,52]. In our case, the desorption process could be the major mechanism of initial burst release because of the attachment of oil to the surface. As ethanol was also included in the release medium, that facilitated the quick release of oil attached on the surface or near-surface. The oil contents inside the fibers were steadily released and the possible mechanism could be diffusion and degradation.

### 3.7. Antioxidant Activity

An elevated level of reactive oxygen species (ROS) or their slow elimination from the body leads to oxidative stress. These ROS (e.g., superoxide anion, hydrogen peroxide, hydroxyl radical, and lipid peroxides) are the byproduct of aerobic metabolism but their level can be increased under pathophysiological conditions. ROS attack biological macromolecules such as membrane lipids, nucleic acids, carbohydrates, and proteins resulting in injury to cells [53,54].

The OEO acts as reducing agent thus significantly decreasing the level of ROS. Chemical constituents like phenols (thymol, carvacrol) and monocyclic hydrocarbons (terpinolene, R-terpinene, and *γ*-terpinene) are active compounds possessing antioxidant activity [9,55].

**Figure 6:**
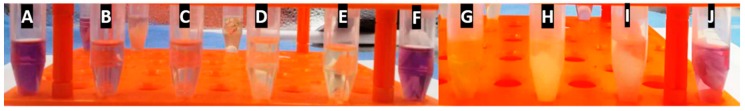
Colour changes (**A**–**J**); DPPH, OEO 1 mg, OEO 2 mg, Ascorbic acid (AA) 1 mg, AA 2 mg, Water, PLCL/SF/7.5% OEO, PLCL/SF/5% OEO, PLCL/SF/2.5% OEO, PLCL/SF.

**Figure 7:**
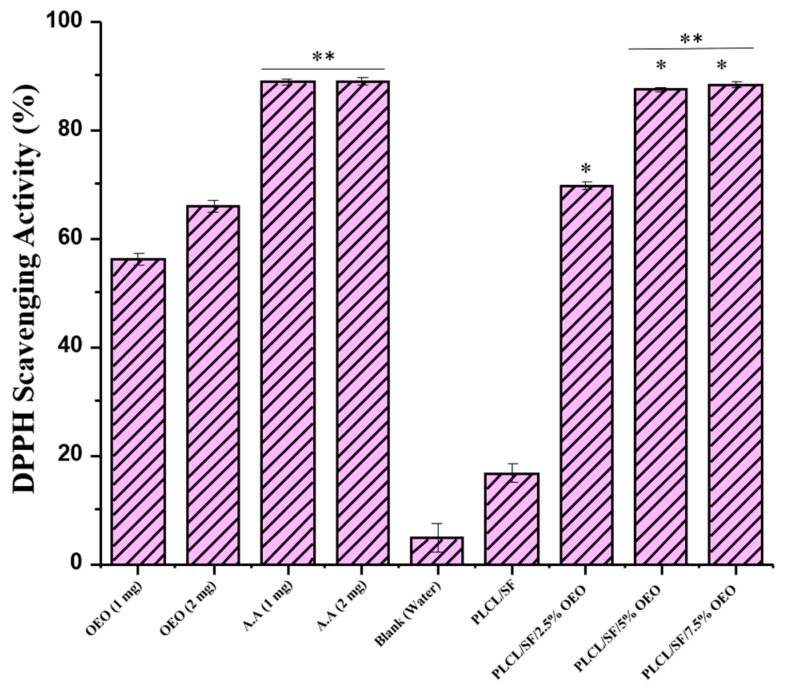
2,2-diphenyl-1-picryl-hydrazyl-hydrate (DPPH) scavenging activity of various samples for 30 min time. (* = significant difference in comparison with PLACL/SF, ** = non-significant with each other).

DPPH is a stable radical usually used in determining Antioxidant activity of a compound. Antioxidant compounds can easily scavenge the radicals of DPPH. Antioxidant agents act as a donor of electrons and hydrogen atoms through which scavenging activity takes place. Moreover, as shown in Figure 6, this reaction leads to colour changes from purple, induced by DPPH, to yellow. [56]. Figure 7 shows the antioxidant activity of various groups after 30 min. In this experiment, NF membranes with various OEO concentrations (2.5%, 5%, and 7.5%) were tested for their antioxidant potential and the results were compared with NF membranes without OEO, pure OEO, and AA.

According to the results, the antioxidant activity of the PLCL/SF NF membrane was noted as 16.7 ± 1.9. This antioxidant activity can be attributed to SF containing amino acid like tyrosine and tryptophan which contain phenolic side chains [57]. The scavenging activity for OEO loaded NF membranes (2.5%, 5%, and 7.5%) remained 69.8 ± 0.8, 87.5 ± 0.3 and 88.4 ± 0.5 respectively. This result shows that OEO loaded NF membranes have antioxidant activity significantly different (*p* < 0.05) from NF without OEO, strongly indicating the association of OEO with antioxidant activity. Moreover, the scavenging activity of NF membranes with 5% and 7.5% OEO have antioxidant activity comparable to that of AA. OEO loaded NF membranes have higher antioxidant activity compared to pure OEO, which confirms the claim that the therapeutic potential oil OEO can be enhanced by the electrospinning technique because of the special features of the electrospun NF membrane.

**Figure 8:**
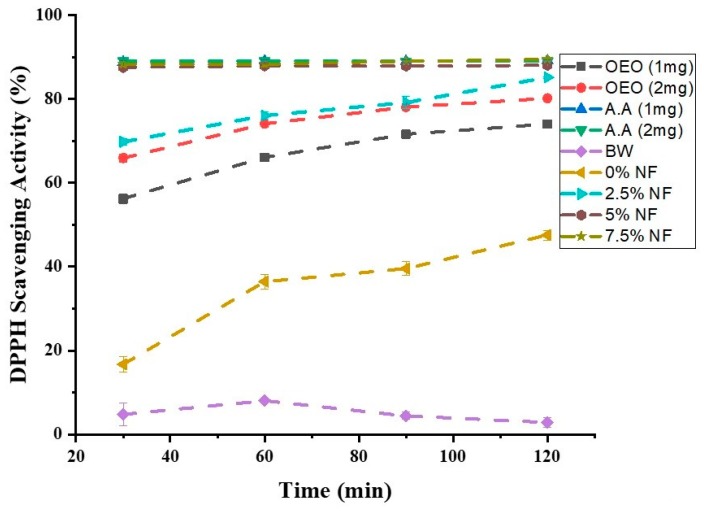
Time-dependent Antioxidant Activity of different groups.

The experiment was also extended to check the time-dependent scavenging activity. Figure 8 shows that the antioxidant activity of various tested groups went up with time. But AA and NF membranes with 5% and 7.5% OEO contents showed complete scavenging activity within the first 30 min, arguing in favour of their maximum potential.

### 3.8. Anti-Tumor Activity

**Figure 9:**
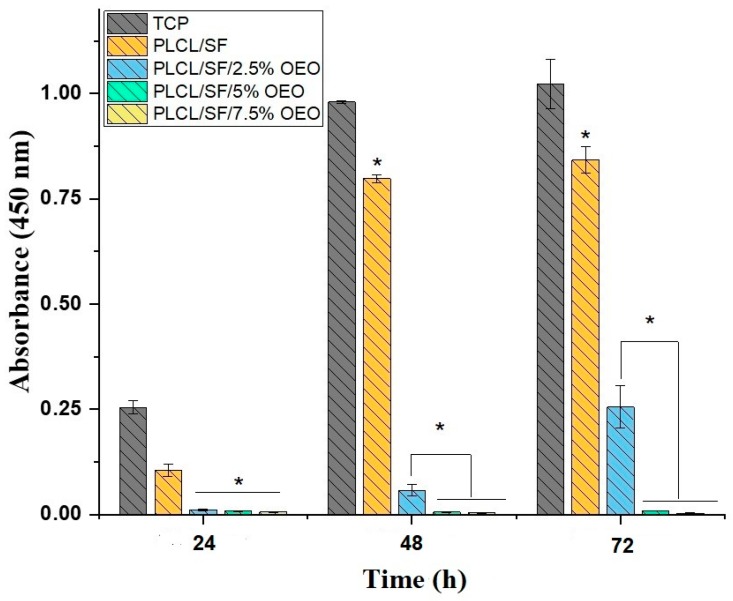
Cell counting Kit-8 (CCK-8) result of 4T1 cell line proliferation on NF membranes (* = significant difference; *p* < 0.05 n = 3).

**Figure 10:**
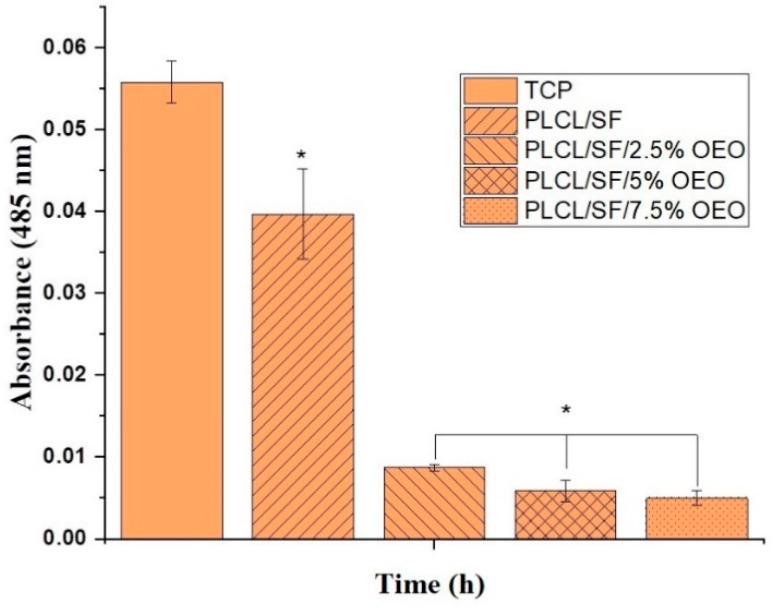
Calcein absorbance after 48 h culture of 4T1 cell line on NF membranes. (* = significant difference; *p* < 0.05 n = 3).

The anti-tumor activity of the electrospun NF membrane loaded with various concentrations of OEO was analysed by a CCK-8 cell viability assay. Figure 9 indicates the absorbance after 24, 48 and 72 h. According to the results, after 24 h, a statistically significant (*p* < 0.05) anti-proliferative activity was seen in NF membranes containing OEO with PLCL/SF. However, after 48 h, we observed some proliferation of cells on the NF membrane with 2.5% OEO which tended to increase with time. However, this activity was significantly different from the PLCL/SF NF membrane. The NF membranes with 5% and 7.5% OEO contents showed very strong antiproliferative activity throughout 72 h of study. Figure 10 shows the absorbance of Calcein dye used for the detection of viable cells. The results of the Calcein assay confirmed the results of CCK-8 assay that the viability of cells dramatically decreased when cultured on OEO loaded NF membranes.

Although OEO varies in composition, depending upon its origin and extraction method, in this study, OEO was used in which the carvacrol contents were about 78%, followed by thymol as a second major component, constituting about 7% according to the manufacturer’s information. Both compounds are associated with anti-cancer activities [30,32,33]. The higher antiproliferative activity can be related to the higher content of carvacrol and thymol as well as a large surface area for drug action. There is no available literature to compare the result of OEO loaded into NF for its antitumor activity; however, many in vitro cell lines model were studied for anti-tumor activities. Marrelli et al. studied the colon carcinoma cell line (LoVo) and got 58.39% of inhibition after 24 h [34]. Similarly, Begnini et al. tried human breast adenocarcinoma (MCF-7) and human colon adenocarcinoma (HT-29) cell lines and achieved 60.8% and 48.9% of inhibitions, respectively [33]. Similarly, human lung adenocarcinoma epithelial (A549) was also investigated for antiproliferative activity suggesting that OEO interferes with the mitosis of cells and thus causes cell death [58]. All these and many other studies indicate the potential of OEO against various cancer cell lines. In this study, we used the 4T1 cell line and proved that OEO has a very strong inhibitory activity which is more accelerated by the electrospun drug delivery and activity system.

## 4. Conclusions

In this study, we successfully loaded OEO into the blend of PLCL/SF to get nanofibers through electrospinning. Loading of OEO was confirmed by FTIR and TGA. LC-MS data revealed that OEO continued to release from polymers for more than 48 h. The morphological and mechanical properties of the NF membrane are suitable for use in biomedical applications. To demonstrate the therapeutic potential of the material, we examined the anti-oxidant and anti-tumor activities. A DPPH assay and In-vitro cell (4T1) culture assay revealed that the material is not only anti-oxidant but also has a cytotoxic effect against the tumor cell line. This work confirms the possibility of incorporating OEO into the blend of PLCL/SF to produce nanofibers to achieve potential biomedical applications, especially for the post-surgical treatment of tumors to avoid recurrence of the disease.

## Figures and Tables

**Table 1:** Mean pore diameter and porosity of various Nanofibrous (NF) membranes with Standard deviation (SD) values.

Name	Mean Pore Diameter ± SD (nm)	Porosity (%)
Poly (l-lactic acid-*co*-e-caprolactone)/Silk Fibroin (PLCL/SF)	1201.9 ± 356.7	51.6 ± 2.9
PLCL/SF/2.5% Oregano Essential Oil (OEO)	1588.07 ± 487.4	61.3 ± 2.6
PLCL/SF/5% OEO	1783.3 ± 731.6	63.7 ± 2.5
PLCL/SF/7.5% OEO	1504.1 ± 284.4	68.6 ± 2.6

**Table 2:** Thermogravimetric Analysis of SF, PLCL and Electrospun Membranes Loaded With OEO.

Samples	*T*_onset_ (°C)	*T_end_*_set_ (°C)	Mass Loss (%)	*T*_onset_ (°C)	*T_end_*_set_ (°C)	Mass Loss (%)	*T*_onset_ (°C)	*T_end_*_set_ (°C)	Mass Loss (%)	*T*_onset_ (°C)	*T_end_*_set_ (°C)	Mass Loss (%)
SF	50.6	120.8	8.3	202.1	244.3	2.6	244.2	350.3	36.6	350.3	600	18.3
PLCL	245.6	436.1	96.5	-	-	-	-	-	-	-	-	-
PLCL/SF/0% OEO	100	250	1.24	250	400	77.9	400	452	3	452	600	2.71
PLCL/SF/2.5% OEO	100	250	3.5	250	400	76.2	400	452	2.8	452	600	2.6
PLCL/SF/5% OEO	100	250	8.5	250	400	69.3	400	452	3.3	452	600	2.5
PLCL/SF/7.5% OEO	100	250	10.7	250	400	66.3	400	452	3.1	452	600	2
